# The Underdiagnosis of Chronic Kidney Disease in Patients with a Documented Estimated Glomerular Filtration Rate and/or Urine Albumin–Creatinine Ratio in Germany

**DOI:** 10.3390/medicina61050843

**Published:** 2025-05-02

**Authors:** Karel Kostev, Martina Lang, Sven-Oliver Tröbs, Sophia Urbisch, Maximilian Gabler

**Affiliations:** 1 IQVIA, Epidemiology, 60549 Frankfurt am Main, Germany; 2 University Hospital, Philipps University of Marburg, 35043 Marburg, Germany; 3 IQVIA, Real World Commercial Analytics, 60549 Frankfurt am Main, Germany; 4 Boehringer Ingelheim Pharma GmbH & Co. KG, Market Access, 55218 Ingelheim, Germany; 5 Boehringer Ingelheim Pharma GmbH & Co. KG, TA CRM Medicine, 55218 Ingelheim, Germany

**Keywords:** chronic kidney disease (CKD), urine albumin–creatinine ratio (UACR), eGFR (estimated glomerular filtration rate), underdiagnosis

## Abstract

*Background and Objectives:* The underdiagnosis of chronic kidney disease (CKD) is a critical issue worldwide. This present study aimed to explore the CKD subpopulation regarding underdiagnosed CKD in individuals with a pathological estimated glomerular filtration rate (eGFR) and/or urine albumin–creatinine ratio (UACR) values in Germany. *Materials and Methods:* This analysis used data from the IQVIA^TM^ Disease Analyzer database and included adult outpatients with at least two pathological consecutive eGFR and/or UACR values, documented at least 60 days apart between October 2018 and September 2023 in 758 general practices. According to the 2024 KDIGO clinical practical guidelines, CKD was defined based on both eGFR and UACR values. UACR values were used when no pathological eGFR values were documented. The main outcome of the study was the proportion of patients with a documented CKD in the total population as well as in defined subgroups. *Results:* A total of 113,996 CKD patients (mean age: 76.5 (SD: 10.1) years; 60.2% female; 71.5% with mildly to moderately, 21.1% with moderately to severely, and 4.6% with a severely decreased eGFR value; and 1.0% with end-stage kidney disease) were available for analyses. CKD diagnosis was documented in 46.9% of CKD patients and was more frequent in male than in female patients (53.3% versus 42.7%). The highest proportion was observed in patients with heart failure (57.0%), followed by patients with type 2 diabetes (52.7%). In patients without diabetes and heart failure, CKD was documented in 38.2%. The proportion of CKD diagnoses increased with decreasing eGFR values, from 22.0% in patients with nonpathological eGFR but moderately or severely increased UACR to 87.7% in patients with end-stage kidney disease. *Conclusions:* The study provides valuable insights into the subpopulation of underdiagnosed CKD patients among a large patient population. These results underscore the need for improved screening, timely diagnosis documentation, and treatment strategies for CKD, particularly among high-risk populations. Moreover, it raises the need to increase awareness of micro- and macroalbuminuria as diagnostic criteria for CKD independent of eGFR.

## 1. Introduction

Chronic kidney disease (CKD) is a prevalent and progressive condition that affects millions of individuals worldwide. The condition often leads to severe health complications and increased mortality rates [1]. CKD prevalence varies considerably between countries in Europe and also between regions in Germany [2,3]. The estimated prevalence (CKD stages 1–5) in individuals aged between 31 and 82 years ranges from around 10% (Cooperative Health Research in the Region of Augsburg (KORA F4) Study) in Southern Germany to over 20% (Study of Health in Pomerania (SHIP-1)) in Northeast Germany [2]. Stolpe et al. reported an estimated prevalence (CKD stages 3–5) of approximately 10% in individuals aged ≥40 years [4].

Early identification and intervention are critical to mitigating the adverse outcomes associated with CKD. The estimated glomerular filtration rate (eGFR), a measure of kidney function derived from serum creatinine levels, is a primary laboratory measurement for the detection of CKD. The urine albumin–creatinine ratio (UACR) is another important diagnostic tool in the early detection of kidney disease. Patients with nonpathological eGFR values may have microalbuminuria or macroalbuminuria when UACR is increased [5,6]. Despite the availability of eGFR and UACR testing, a significant issue remains with the underdiagnosis of CKD, mainly because CKD is asymptomatic in its early stages. Previous studies have already reported low rates of UACR testing in France (29%) [7] and the US (21%) [8]. A retrospective cross-sectional study based on 448,837 patients in Germany (“InspeCKD”) found that UACR had only been assessed in 0.4% of patients at risk of CKD (defined by an underlying diagnosis of hypertension, type 2 diabetes mellitus, or cardiovascular disease). In addition, less than half of patients had a documented eGFR measurement (45.5%) [9].

These findings indicate CKD diagnosis may frequently be underdiagnosed, as previous studies have also reported [10,11,12].

The underdiagnosis of CKD is an important issue, particularly among individuals exhibiting pathological eGFR and UACR values. For example, Stolpe et al. utilized data collected in 2010 from German CKD research cohorts and registries to conduct a survey with 3305 individuals with prevalent renal insufficiency based on their eGFR values. The authors reported that 68% of study participants were unaware of their CKD. The rate of unawareness was 80% in CKD stages 1/2, 71% in CKD stage 3a, 49% in stage 3b, and more than 30% in CKD stage 4 [4]. In a study published by Wagner et al. investigating hospitalized patients with coronary artery disease, 66% of 158 patients with kidney disease were not aware of their CKD [13]. Low levels of CKD awareness in patients with pathological eGFR values have also been reported in other countries such as the USA and Australia [14].

Recently, Tangri et al. analyzed electronic medical records and claims data from five different countries. Their findings demonstrated the high prevalence of undiagnosed stage 3 CKD among patients with stage 3 CKD—96% in France, 92% in Japan, 84% in Germany, 77% in Italy, and 62–64% in the USA—all predominantly based on eGFR values. The overall prevalence of UACR testing was very low, ranging from 1.8% to 5.5% [15]. Older age, female sex, and no history of diabetes and hypertension were associated with a higher risk of stage 3 CKD underdiagnosis [15].

Sundström et al. estimated the prevalence of CKD across eleven countries using data from digital healthcare systems. The authors reported that more than 65% of CKD patients identified using laboratory criteria did not have a corresponding CKD diagnosis code [16]. Although the upward trend in CKD recognition is a clinically meaningful improvement, nearly half of patients with low eGFR levels remained undiagnosed in the most recent period. The present findings from CURE-CKD point to the urgent need for quality improvement and research at the point of care [17].

CKD and, in particular, undiagnosed CKD, poses a considerable challenge to public health. The condition is often only diagnosed at an advanced stage that has already led to irreversible kidney damage that is likely to progress to end-stage kidney disease, which is associated with a markedly higher cardiovascular risk [18]. Thus, the evidence described above on the high prevalence of undiagnosed CKD among CKD patients indicates a need to improve the care offered to CKD patients. However, current research findings are subject to several limitations that should be acknowledged. First, a substantial proportion of studies were conducted outside of Germany, which does not report any data on measured vs. diagnosed CKD (i.e., [16]), and it may not be possible to extrapolate their conclusions to the German healthcare situation. Second, only a small number of studies analyzed the underdiagnosis of CKD in patients with two pathological eGFR values, and UACR measurements were either absent or hardly available. Furthermore, one study using the KDIGO definition was limited to stage 3 CKD patients [15].

It is crucial to gain more information on the underdiagnosis of CKD in Germany overall as well as across patients in various stages of CKD. The aim of the present study was to estimate the number of undiagnosed CKD patients among patients with pathological eGFR and/or UACR values, stratified by age, sex, eGFR and UACR stage, and relevant comorbidities.

## 2. Methods

### 2.1. Data Source

This analysis used data from the IQVIA^TM^ Disease Analyzer database, which contains case-based information provided by office-based physicians (both GPs and specialists) in Germany. Information is available on patient demographics, drug prescriptions, concomitant medication, comorbid conditions, sick leave, and lab values. In total, 3000 office-based physicians provide information regularly. Analyses conducted in comparison with reference statistics did not indicate any lack of representativeness or validity with respect to the Disease Analyzer database. The database appears to be suitable for pharmacoepidemiologic and pharmacoeconomic studies [19] and has already been utilized in peer-reviewed publications on CKD [20,21].

### 2.2. Ethical Considerations

German law allows the use of anonymous deidentified electronic medical records for research purposes under certain conditions. According to this legislation, it is not necessary to obtain informed consent from patients or approval from a medical ethics committee for this type of observational study that contains no directly identifiable data. The company and authors involved had no access to any identifying information at any time during the analysis of the data.

### 2.3. Study Population and Outcomes

**Figure 1:**
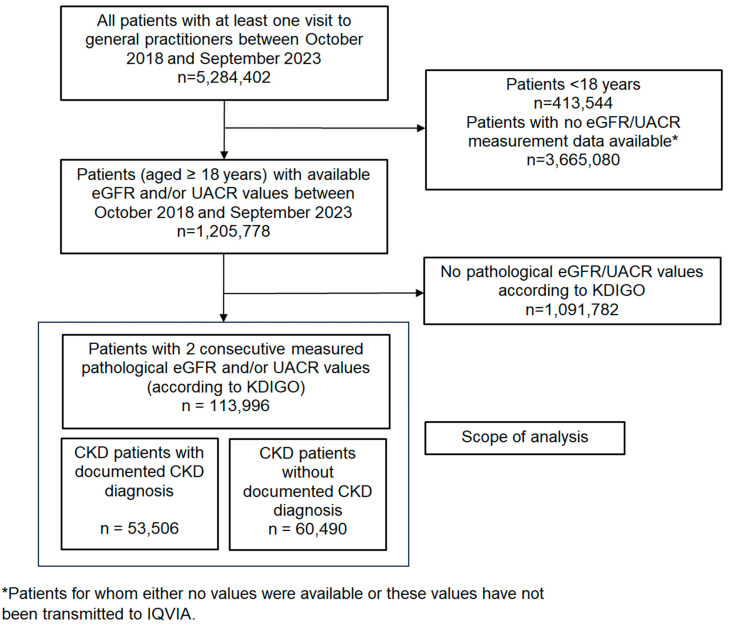
Derivation of the study population.

This study used the electronic medical records of outpatients aged ≥18 years from 882 general practitioner practices (GPs) with documented creatinine values between October 2018 and September 2023. Of these, 758 practices reported eGFR or creatinine measurements, 289 reported UACR measurements, and 256 reported both eGFR/creatinine and UACR measurements. To calculate the eGFR values, the MDRD equation was used (eGFR (mL/min/1.73 m^2^) = 186 × (S.Cr in μmol/L × 0.011312)^−1.154^ × (age)^−0.203^ × (0.742 if female)) [22,23]. The study population consisted of individuals with at least two consecutive pathological eGFR and/or UACR values documented at least 60 days apart, based on the Kidney Disease Improving Global Outcomes (KDIGO) definition of CKD [6]. However, only patients with at least one visit to one of the 758 GPs between October 2021 and September 2023 were included (active patients). The flow chart below describes the scope of the present analysis. Of note, the share of diagnosed and undiagnosed patients is based on the CKD subpopulation and not on the overall patient population (Figure 1).

**Figure 2:**
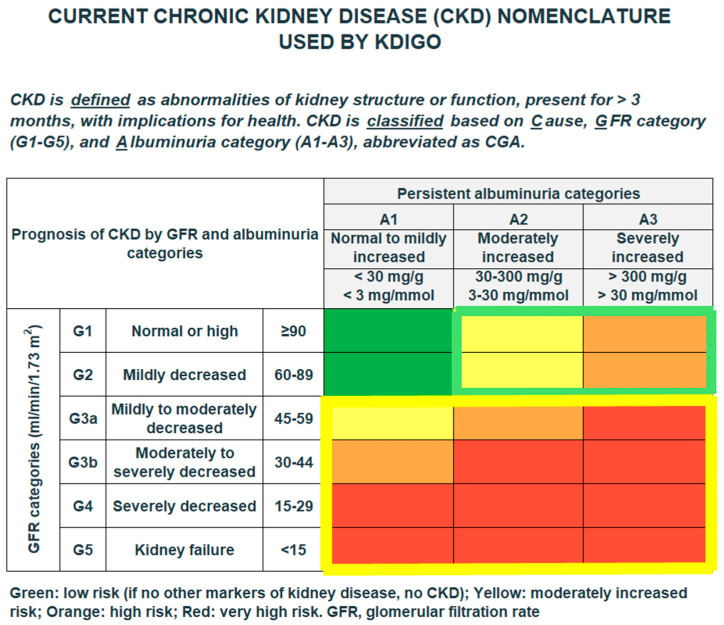
Stages of chronic kidney disease according to current KDIGO 2024 guideline recommendations.

In line with the CKD nomenclature currently used by KDIGO (Figure 2), an eGFR < 60 mL/min/1.73 m^2^ and a UACR ≥ 30 mg/g were considered pathological. According to KDIGO, the eGFR or UACR values have to be pathological as confirmed by consecutive measurements at least 3 months apart. However, many patients have outpatient visits on a quarterly basis and do not strictly adhere to the 90-day interval. It might be the case that the patient receives the next measurement to confirm the value less than 90 days apart in the next quarter. To account for these circumstances, we have chosen an interval of 60 days. It is acknowledged that there are further criteria defining a CKD beyond eGFR and UACR, such as persistent hematuria. Since the classification system is based on eGFR and UACR, eGFR and UACR have been selected to identify CKD patients in the present analysis. Figure 1 illustrates the prognosis of CKD based on the eGFR and UACR categories.

The outcome of the study was the proportion of patients with a documented CKD (ICD-10: N18, N19) in their patient history (from October 2018 to September 2023). For patients with documented CKD, the proportion of ICD-10 codes (N18.8, N18.9, and/or N19) were shown to display the frequency of the use of ICD-10 codes without a defined CKD stage. These codes were only counted when no codes N18.1–N18.5 were documented for the respective patient. Moreover, differences in diagnosis rates between patients whose CKD is diagnosed by the persistence of micro- and macroalbuminuria (G1/G2 + A2/A3) vs. patients whose CKD is identified by an eGFR < 60 (G3/G4/G5 + A1/A2/A3) have been displayed. This approach follows the hypothesis that the awareness of UACR as a diagnostic tool for CKD is much lower than that of eGFR, as suggested by the lower use of UACR measurements by German GPs compared to eGFR [9]. Consequently, the share of undiagnosed CKD patients is assumed to be much higher in CKD patients with an eGFR ≥ 60 and micro- or macroalbuminuria (the CKD diagnosis must be based on UACR) compared to patients with an eGFR < 60 (the CKD diagnosis can be based on eGFR alone, irrespective of UACR).

This prevalence was estimated for the CKD subpopulation overall as well as separately by age group, gender, and the presence of a diagnosis of type 2 diabetes mellitus (ICD-10: E11) and heart failure (ICD-10: I50).

Finally, for patients with at least one confirmed CKD diagnosis documented after the first pathological eGFR and/or UACR value, the time between the first pathological value in the study time and the first documentation of CKD diagnosis was estimated and presented in days with a median value and the 25th and 75th percentiles.

### 2.4. Statistical Analyses

The present cross-sectional study was of a descriptive nature without hypothesis tests. All analyses were conducted using SAS Version 9.4 (AS Institute, Cary, NC, USA).

## 3. Results

### 3.1. Baseline Characteristics

A total of 113,996 patients with at least two pathological consecutive eGFR and/or UACR values documented at least 60 days apart were available for analysis. Table 1 shows the baseline characteristics of the study population. The mean age was 76.5 ± 10.1 years, and 60.2% were female. Nearly half of the population had prevalent type 2 diabetes mellitus (47.6%) and 29.7% had diagnosed heart failure. A total of 71.5% had mildly to moderately decreased kidney function (eGFR ≥ 60 mL/min/1.73 m^2^); 21.1% had moderately to severely (eGFR 30–59 mL/min/1.73 m^2^) and 4.6% had severely decreased eGFR values (eGFR 15–29 mL/min/1.73 m^2^). End-stage kidney disease (kidney failure, eGFR ≤ 15 mL/min/1.73 m) was observed in only 1.0% of the study patients. Only 1.7% of the patients had increased UACR measures without pathological eGFR values. In the total population, UACR measurement was documented in 4.2% (1.0% with UACR stage A1 (<30 mg/g) and 2.2% with UACR stage A2 (30–300 mg/g), and 1.0% with UACR stage A3 (>300 mg/g)) (Table 1).

### 3.2. Diagnosed and Undiagnosed CKD

**Figure 3:**
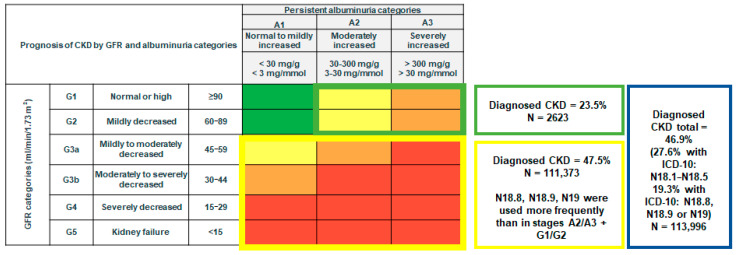
CKD diagnosis in patients with at least two pathological consecutive eGFR and/or UACR values.

Of the 113,996 study patients, 53,506 (46.9%) had documented CKD (53.1% had no CKD diagnosis). Solely ICD-10 Codes N18.8, N18.9, or N19 (unspecified kidney failure) were used in 19% of the study population. The prevalence of CKD documentation was higher in stages G3A to G5 (47.5%) compared to stages A2/A3 + G1/G2 (23.5%) (Figure 3).

### 3.3. CKD by Age, Sex, Comorbidities, and eGFR

**Figure 4:**
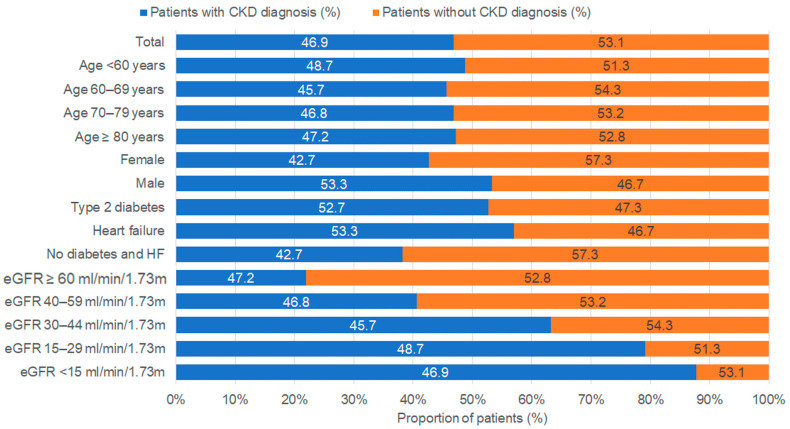
Prevalence of CKD diagnosis and underdiagnosis in patients with at least two pathological consecutive eGFR and/or UACR measures by age, sex, comorbidities, and eGFR.

The prevalence of diagnosed and undiagnosed CKD among patients in the CKD subpopulation by age group, sex, *comorbidities*, and eGFR is shown in Figure 4. In total, 46.9% were diagnosed with CKD. This proportion was similar across all age groups. Moreover, the diagnosis of CKD was more often established in males (53.3% versus 42.7%) (Figure 4).

The proportion of patients with a documented CKD diagnosis was higher among patients with comorbidities. In total, 57.0% of heart failure patients and 52.7% of T2D patients had an established CKD diagnosis, while in patients with neither T2D nor heart failure, CKD was documented in only 38.2% of patients (Figure 4). The proportion of CKD diagnoses increased with decreasing eGFR values, from 22.0% in patients with normal eGFR (>90 mL/min/1.73 m) but moderately or severely increased UACR (≥30 mg/g) to 87.7% in patients with end-stage kidney disease (eGFR < 15 mL/min/1.73 m) (Figure 4).

### 3.4. Time Between Pathological eGFR/UACR Measure and CKD Diagnosis Documentation

**Figure 5:**
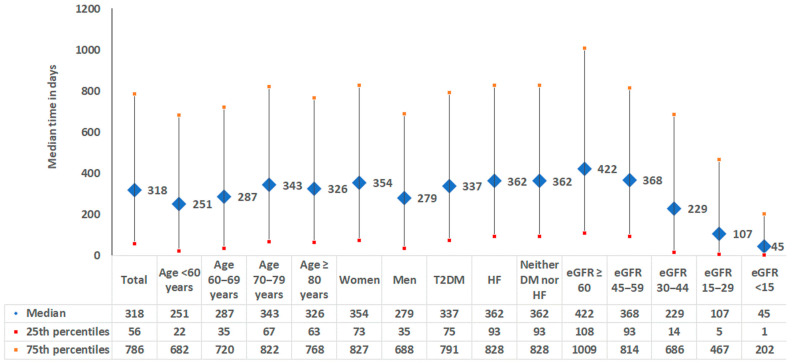
Time between pathological eGFR/UACR measure and first confirmed CKD diagnosis documentation (n = 17,471 patients). T2DM = type 2 diabetes mellitus, HF = heart failure; eGFR values are measured in mL/min/1.73 m.

The median time between the first pathological eGFR or UACR measures and subsequent documented CKD diagnosis in the total population was 318 days; whereby, this time markedly decreased from 422 days in patients with eGFR ≥ 60 mL/min/1.73 m and UACR ≥ 30 mg/g to 45 days in patients with end-stage kidney disease (<15 mL/min/1.73 m). Moreover, this duration increased from 251 days in the age group < 60 years to 326 days in patients aged ≥ 80 years (Figure 5).

## 4. Discussion

In this retrospective study based on 113,996 patients treated in German general practices, we found that only 47% of patients with evident pathological eGFR and/or UACR values had a documented diagnosis of CKD. These findings demonstrate a high rate of undiagnosed CKD in Germany. In patients with CKD driven exclusively by elevated UACR values and normal eGFR, the diagnosis rate is considerably lower than the diagnosis rate observed in patients with reduced eGFR (47.5%), at just 22%. The disparity in documentation rates among patients with and without comorbidities such as diabetes mellitus and heart failure is a further indication that CKD might often be overlooked, especially in patients without these prominent risk factors.

In a study by Tangri et al., the proportion of patients with stage 3 CKD without an established CKD diagnosis in Germany was 84% [15], compared to 37–59% in the present investigation. This disparity is due to differences in the study timeline definitions. Specifically, Tangri et al. defined underdiagnosis as the absence of an ICD diagnosis code for CKD (any stage) any time before and up to 6 months after the second qualifying eGFR measurement [15]. In the present study, a CKD diagnosis at any point throughout the 5-year study period was acceptable (and diagnosis could also be based on UACR measurements). In line with Tangri et al., we found that the proportion of undiagnosed CKD was higher in women and patients without a diabetes diagnosis. The diagnosis rate in the present study was particularly low in patients with nonpathological eGFR scores but elevated UACR values. This mirrored the findings of Tanaka et al., who analyzed data from clinical practice in Japan [24].

Another noteworthy aspect of the study is the sex disparity in the CKD diagnosis rates. The higher share of undocumented CKD among female CKD patients raises questions about potential social factors contributing to this difference. Previous research has indicated that the prevalence of CKD is higher in women than in men [25,26]. However, men have a higher risk of progression to end-stage renal disease than women [27]. This could partly explain the higher diagnosis rates in males, as HCPs have a higher awareness of renal and cardiovascular risk in men than women. This disparity also underscores the need for further investigation into sex-specific factors influencing CKD diagnosis and progression.

The findings of this study have several important implications for clinical practice and healthcare policy. There is a clear need for improved screening and early detection strategies for CKD, particularly among patients with risk factors such as diabetes and heart failure. The regular monitoring of kidney function and albuminuria in these patients, as recommended in clinical guidelines [6], can facilitate early diagnosis and intervention, potentially slowing the progression of CKD and reducing the risk of end-stage kidney disease (KDIGO 2024). While UACR is an established cardiovascular risk marker [28,29,30], the higher share of CKD underdiagnosis in patients whose CKD is driven by elevated UACR values emphasizes the need for the medical community to focus more closely on UACR in the context of CKD, i.e., to link UACR values to a CKD diagnosis. This is particularly important because even with nonpathological eGFR values, patients with persistent micro- or macroalbuminuria have a moderate to high risk of progression to end-stage kidney disease [6].

At the same time, the German Federal Joint Committee’s (G-BA) Disease Management Program (DMP) for T2D does not require UACR measurement, although it does provide for eGFR checks. Moreover, measuring UACR in patients reduces the bonus (known as the “Wirtschaftlichkeitsbonus”, or economic efficiency bonus) paid to outpatient doctors.

These factors may also explain the infrequent use and low awareness of UACR. The issue could therefore be addressed by healthcare policy changes, e.g., by adding UACR as a mandatory measurement to the T2D DMP and/or by introducing an exception code (“Ausnahmekennziffer”) for UACR measurement for all patients so that measuring UACR does not reduce the doctor’s bonus. For this bonus, there are only some exemptions regarding the measurement of albumin for type 2 diabetes and/or CKD patients with creatinine clearance < 25 mL/min, but not for the measurement of creatinine which is required to calculate the UACR.

However, the underdiagnosis of CKD could also stem from other causes, including a lack of awareness among healthcare providers or the misinterpretation of the early indicators of kidney dysfunction. General practitioners are responsible for a wide range of disorders and health issues including infections, injuries, endocrine, cardiovascular, respiratory, and mental disorders. CKD is only one of these disorders. In addition, there was no specific treatment for CKD for more than 20 years (beyond drugs for the treatment of underlying diseases such as T2D or hypertension). Therefore, there was no benefit in diagnosing CKD and thus no incentive for HCPs to do so. With the approval of SGLT2i for the treatment of CKD, the situation has changed markedly. Large phase III clinical trials with the sodium–glucose cotransporter-2 inhibitors (SGLT2i) empagliflozin, dapagliflozin, and canagliflozin have shown that this drug class can effectively reduce the risk of CKD progression, end-stage kidney disease, and renal-related mortality [29,30,31,32,33]. The beneficial renal-protective effects of SGLT2is have been demonstrated across a broad spectrum of CKD stages, supporting their early use in CKD patients. This means that HCPs can now rely on an effective and well-tolerated drug class to treat their CKD patients and may be more likely to diagnose the condition as a result.

According to the current guideline [6], treatment should be initiated promptly after diagnosis of CKD. The current diagnostic gap therefore implies that a relevant number of patients might not be receiving optimal care and treatment for their CKD. As CKD is a progressive disease, early treatment is crucial to delay disease progression and, ideally, to prevent patients from developing end-stage kidney disease. Concretely, KDIGO recommends starting renin–angiotensin system inhibitors (RASis) for individuals with CKD and moderately to severely increased albuminuria with or without diabetes [34]. Furthermore, KDIGO recommends SGLT2i treatment for patients with T2D and CKD [6].

The study highlights a significant correlation between CKD and comorbidities such as type 2 diabetes and heart failure. The proportion of CKD diagnoses was highest among patients with heart failure, followed by those with type 2 diabetes (47.6% and 29.7%, respectively). These findings are consistent with the recognition of diabetes and heart failure as major risk factors for CKD and trigger physicians’ awareness of concomitant diseases like CKD. Diabetes often results in kidney damage leading to diabetic nephropathy, a leading cause of CKD, while heart failure can cause cardiorenal syndrome [35,36,37,38]. This emphasizes the importance of monitoring eGFR and UACR closely in such high-risk patients to ensure the early detection and management of CKD. Micro- or macroalbuminuria is a sign of endothelial damage and a well-established cardiovascular risk factor. Consequently, patients with these conditions require adequate care and treatment. Although patients with comorbidities have higher CKD diagnosis rates than those without, almost half of these patients still go undiagnosed (47% T2DM/CKD patients, 43% HF/CKD patients). In view of this, the importance of increasing awareness of both CKD diagnostic markers—eGFR and UACR—is clear, especially for patients with comorbidities such as T2DM and HF [39,40].

The median time of 237 days between the first pathological eGFR or UACR measurement and subsequent CKD diagnosis reveals a significant delay in diagnosing CKD. This delay is particularly concerning as the early management of CKD is crucial in slowing disease progression and preventing complications. Our study demonstrates that this delay is inversely related to the severity of eGFR decline, with the median time decreasing from 422 days in patients with eGFR ≥ 60 mL/min/1.73 m and UACR ≥ 30 mg/g to 45 days in those with end-stage kidney disease. This suggests that more severe kidney impairment prompts quicker clinical action, yet it also indicates a potential gap in the timely management of patients with less severe kidney dysfunction. The significant diagnostic delay observed in the study, even in end-stage kidney patients, highlights the necessity for timely follow-up and the reevaluation of patients with abnormal kidney function. Healthcare providers should be aware of the importance of not only conducting initial tests but also of ensuring prompt action and further evaluation when pathological results are identified.

Retrospective primary care database analyses are generally limited by the completeness of their data. We included all patients with documented eGFR and/or UACR values in our study. These values were only measured in a portion of the individuals treated in general practices. Another potential limitation of our study is that some diagnoses may have been coded incorrectly or misclassified by the attending physician. Furthermore, the database used includes neither data on the socioeconomic status of patients, which would facilitate more comprehensive analyses, nor data from hospitals or specialists such as nephrologists.

The strengths of this study include the large patient sample (113,996 patients) and the inclusion of ICD10 coding as well as UACR and eGFR measurements over a significant timeframe, allowing for a comprehensive assessment of CKD in the German healthcare system. Furthermore, our study utilized a database whose reliability has been validated in several medical studies.

## 5. Conclusions

This study provides valuable insights into the subpopulation of underdiagnosed CKD patients among a large German patient population as well as in defined subgroups (i.e., individuals with diabetes, heart failure, different age groups, and different CKD severity stages). These results underscore the need for improved screenings, timely diagnosis documentation, and prompt initiation of treatment for CKD, particularly among high-risk populations. Moreover, the analysis revealed the need to increase awareness of micro- and macroalbuminuria as diagnostic criteria for CKD independent of eGFR.

## Figures and Tables

**Table 1:** The baseline characteristics of the study population.

Variable	N (%) or Mean (SD)
N	113,996
Age (mean, SD)	76.5 (10.1)
Age group (N, %)	
Age <60 years	7414 (6.5)
Age 60–69 years	18,072 (15.9)
Age 70–79 years	37,146 (32.6)
Age ≥ 80 years	51,364 (45.1)
Sex (N, %)	
Women	68,683 (60.2)
Men	45,413 (39.8)
Comorbidities (N, %)	
Type 2 diabetes mellitus	54,278 (47.6)
Heart failure	33,846 (29.7)
Neither diabetes mellitus nor heart failure	44,017 (38.6)
eGFR/UACR category (N, %)	
eGFR not available; UACR ≥ 30 mg/g	652 (0.6)
eGFR ≥ 90 mL/min/1.73 m–UACR ≥ 30 mg/g	813 (0.7)
eGFR 60–89 mL/min/1.73 m–UACR ≥ 30 mg/g	1158 (1.0)
eGFR 45–59 mL/min/1.73 m	81,543 (71.5)
eGFR 30–44 mL/min/1.73 m	24,044 (21.1)
eGFR 15–29 mL/min/1.73 m	5258 (4.6)
End-stage kidney disease (eGFR < 15 mL/min/1.73 m)	528 (0.5)
UACR < 30 mg/g	1119 (1.0)
UACR 30–300 mg/g	2470 (2.2)
UACR > 300 mg/g	1178 (1.0)

## Data Availability

The data and the code used for this study are available from the corresponding author upon request.
